# Comparison of the Immune Responses Induced by Chimeric Alphavirus-Vectored and Formalin-Inactivated Alum-Precipitated Measles Vaccines in Mice

**DOI:** 10.1371/journal.pone.0010297

**Published:** 2010-04-22

**Authors:** M. Jeff Bergen, Chien-Hsiung Pan, Catherine E. Greer, Harold S. Legg, John M. Polo, Diane E. Griffin

**Affiliations:** 1 Graduate Program in Immunology, Johns Hopkins University School of Medicine, Baltimore, Maryland, United States of America; 2 W. Harry Feinstone Department of Molecular Microbiology and Immunology, Johns Hopkins Bloomberg School of Public Health, Baltimore, Maryland, United States of America; 3 Novartis Vaccines and Diagnostics, Cambridge, Massachusetts, United States of America; Federal University of São Paulo, Brazil

## Abstract

A variety of vaccine platforms are under study for development of new vaccines for measles. Problems with past measles vaccines are incompletely understood and underscore the need to understand the types of immune responses induced by different types of vaccines. Detailed immune response evaluation is most easily performed in mice. Although mice are not susceptible to infection with wild type or vaccine strains of measles virus, they can be used for comparative evaluation of the immune responses to measles vaccines of other types. In this study we compared the immune responses in mice to a new protective alphavirus replicon particle vaccine expressing the measles virus hemagglutinin (VEE/SIN-H) with a non-protective formalin-inactivated, alum-precipitated measles vaccine (FI-MV). MV-specific IgG levels were similar, but VEE/SIN-H antibody was high avidity IgG2a with neutralizing activity while FI-MV antibody was low-avidity IgG1 without neutralizing activity. FI-MV antibody was primarily against the nucleoprotein with no priming to H. Germinal centers appeared, peaked and resolved later for FI-MV. Lymph node MV antibody-secreting cells were more numerous after FI-MV than VEE/SIN-H, but were similar in the bone marrow. VEE/SIN-H-induced T cells produced IFN-γ and IL-4 both spontaneously *ex vivo* and after stimulation, while FI-MV-induced T cells produced IL-4 only after stimulation. In summary, VEE/SIN-H induced a balanced T cell response and high avidity neutralizing IgG2a while FI-MV induced a type 2 T cell response, abundant plasmablasts, late germinal centers and low avidity non-neutralizing IgG1 against the nucleoprotein.

## Introduction

Measles remains a significant cause of morbidity and mortality in young children, particularly in sub-Saharan Africa and Asia [1]. Because there is no animal reservoir and an efficacious live-attenuated virus vaccine (LAV) is available, eradication has been considered by global health organizations [1]–[3]. However, LAV is neutralized by passively acquired maternal antibody and cannot be administered effectively during the first months of life, thus both complicating vaccine delivery with a need for an additional health care visit and creating a variable window of susceptibility to measles prior to vaccination [4], [5]. Administration of larger amounts of LAV to bolster vaccine virus replication in the face of maternal antibody resulted in an unexpected late increase in mortality [6], [7]. Current approaches to improving measles vaccine coverage include aerosol delivery of LAV [8] and development of a new measles vaccine able to induce protective immunity in children younger than 6 months of age [9]–[14]. Previous experience with an inactivated vaccine that primed for more severe disease requires a better understanding of the immune responses to measles vaccines of different types before developing a new measles vaccine.

Measles virus (MV) is a member of the *Paramyxoviridae* family in the Morbillivirus genus and encodes 6 structural proteins, including 2 surface glycoproteins, hemagglutinin (H) and fusion (F). Non-envelope structural proteins include nucleocapsid (N), matrix (M) and the replicase proteins, large and phosphoprotein. MV was isolated in 1954 [15] and the first measles vaccines were developed by the early 1960s [16]–[22]. Similar to the polio vaccines developed a decade earlier, two strategies were employed- virus attenuation and inactivation. Both LAV and an alum-precipitated, formalin-inactivated MV vaccine (FI-MV) were licensed in 1963. Subsequently, it was observed that some individuals immunized with FI-MV were not protected from MV infection, despite previous seroconversion, and were at risk for enhanced disease, termed atypical measles, characterized by high fever, unusual petechial rash and pneumonitis [23]–[31]. Despite progress, the immunologic basis for atypical measles remains incompletely understood [32]–[34].

The efficacy of measles vaccines is highly dependent on the ability to induce high-titer, long-lived neutralizing antibody, as occurs after natural infection [35]. Infection induces antibody against most viral proteins [36], but protection correlates with the level of neutralizing antibody that is directed primarily against H and to a lesser extent F [14], [37]–[42]. After FI-MV immunization, antibody titers waned quickly. Two-and-a-half years after receiving a 3-dose course, over 40 percent of children no longer had protective levels of antibody [23]. FI-MV also induced short-lived, low-avidity MV-specific immunoglobulin G (IgG) in rhesus macaques that were then prone to atypical measles on challenge [33], [34]. Studies of vaccinated mice offer the opportunity to examine the nature of the immune response to FI-MV in more detail.

Because LAV does not replicate in mice, this vaccine cannot be used for comparative studies. However, one promising strategy for new vaccine development is the use of alphavirus replicon particles that can be studied in mice [43]. These vaccines contain the alphavirus nonstructural genes, the 5′ and 3′ *cis*-active replication sequences and the subgenomic promoter that directs expression of a heterologous gene [44]. The replicon RNA is then packaged into virus-like particles by providing the structural protein in *trans* [45]–[47]. Several different alphaviruses are being developed as vectors for a variety of vaccine antigens [48]–[50]. These vaccines undergo only a single round of replication and circumvent the problem of interference due to passively acquired maternal antibody because the alphavirus particles will not be neutralized by pre-existing antibody to the heterologous antigen [51]. In addition, these vaccines have intrinsic adjuvant activity that has only been partially characterized [52]–[54].

We have developed a chimeric alphavirus replicon vaccine [48] utilizing the nonstructural genes from Venezuelan equine encephalitis virus as a replicon expressing the MV H protein packaged with Sindbis virus structural proteins to produce the replicon particle vaccine VEE/SIN-H. This vaccine has recently been shown to induce protective immunity in rhesus macaques [55].

To better understand the nature of the non-protective immune response generated by FI-MV, we have used a mouse model to compare the immune responses to FI-MV with the immune response to VEE/SIN-H.

## Results

### MV-specific antibody responses after vaccination

**Figure 1:**
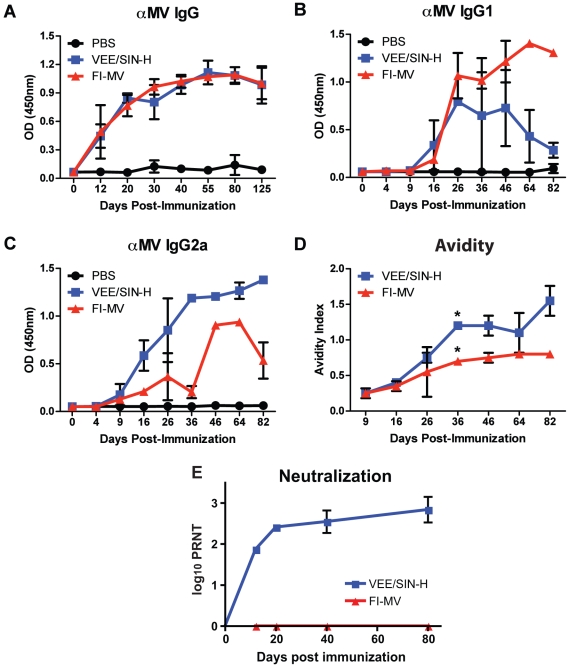
Measles virus-specific antibody response to immunization. Sera collected from individual mice immunized with VEE/SIN-H or FI-MV were assessed for quantity and quality of MV-specific antibody. MV-specific total IgG (**A**), IgG1 (**B**) and IgG2a (**C**) were measured by EIA. Avidity of MV-specific antibody was evaluated by a modified EIA and data are presented as an avidity index (**D**). Fifty percent plaque reduction neutralization titers (PRNT) for the Chicago-1 strain of MV on Vero cells are expressed as geometric means (**E**). Data points represent the mean +/- S.D. of three individual mice. (* *P*<0.05; Student's *t* test)

Although mice are not susceptible to infection with MV or LAV, they offer a well-characterized animal model for evaluating immunogenicity of non-replicating vaccines [9], [11], [13], [56]–[60]. Mice were immunized subcutaneously with single doses of FI-MV or VEE/SIN-H. MV-specific IgG was measured by enzyme immunoassay (EIA) using a measles virus-infected Vero cell lysate (MVL) as antigen (Fig. 1). The time course and magnitude of the IgG response to FI-MV and VEE/SIN-H were similar (Fig. 1A). However, the isotypes were different. FI-MV induced primarily IgG1 antibody, suggesting type 2 T cell help (Fig. 1B), while VEE/SIN-H elicited mostly IgG2a antibody, suggestive of type 1 T cell help (Fig. 1C). MV-specific IgG3 was not detected for either group (data not shown). Antibody avidity steadily improved in VEE/SIN-H–immunized mice and at day 36 was significantly higher (*p*<0.05) than that of antibody induced by FI-MV and remained higher through day 82 (Fig. 1D). Plaque reduction neutralization assays showed geometric mean titers >1∶200 by day 20 and >1∶600 on day 80 for serum from VEE/SIN-H-immunized mice, but no neutralization by serum from FI-MV mice (Fig. 1E).

### VEE/SIN-H and FI-MV initiate germinal center reactions with similar magnitude, but different kinetics

**Figure 2:**
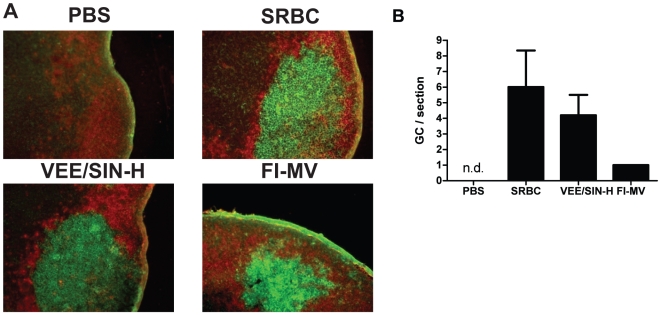
Germinal center formation after immunization. Popliteal draining lymph nodes were harvested at day 7 after injection with VEE/SIN-H, FI-MV, SRBCs or PBS. Cryosections (10µm) were stained with PNA-FITC for GC B cells (green) and with PE-conjugated antibody to IgD for follicular B cells (red). Representative images are 200× magnification (**A**). GCs detected by histology were enumerated and presented as the mean +/− S.D. of at least 5 sections from one mouse (n.d. = none detected) (**B**).

**Figure 3:**
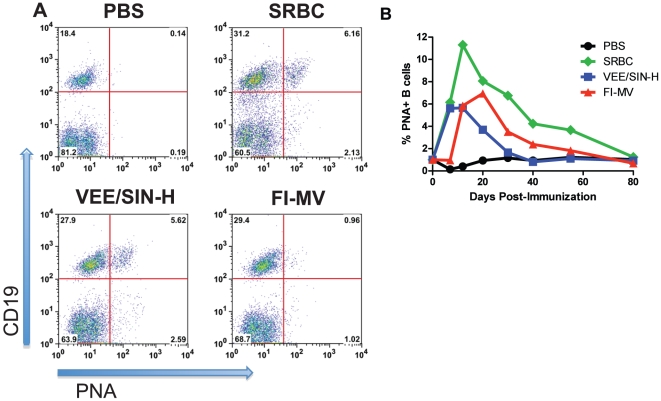
Kinetics of the germinal center response after immunization. Cells from popliteal draining lymph nodes harvested at various times after immunization with VEE/SIN-H, FI-MV, SRBCs (positive control) or PBS were analyzed for GC B cells by flow cytometry. Cells were stained with antibody to CD19 and PNA at day 7 (**A**) and periodically over an 80-day time course (**B**). All flow cytometry data points represent values from cells pooled from 2 mice.

To determine whether the lack of production of avid antibody against MV after FI-MV was due to a deficit in the formation of germinal centers (GCs), dLNs were evaluated for numbers of GCs (Fig. 2) and GC B cells (PNA^+^CD19^+^) (Fig. 3) after immunization. Control mice were immunized with sheep red blood cells (SRBCs), a complex T-dependent antigen that induces a robust GC reaction [61], [62], or with PBS. Histological examination showed few GCs at day 7 after FI-MV immunization compared to immunization with SRBC or VEE/SIN-H (Fig. 2B) and the GCs observed were not well formed (Fig. 2A). Flow cytometry was used to quantify peanut agglutinin (PNA)^+^ B cells (Fig. 3A). On day 7, few PNA^+^ CD19^+^ B cells were present in the dLNs of FI-MV–immunized mice (0.96%), similar to the PBS control (0.14%), while the percentage of GC B cells in the dLNs of VEE/SIN-H (5.6%) was similar to SRBC-immunized mice (6.16%).

To determine the kinetics of the GC reaction in the dLNs, mice were evaluated at multiple times after immunization (Fig. 3B). GC B cells were most numerous 7–12 days after immunization for VEE/SIN-H (5.65%) and SRBC (11.3%) but peak responses were delayed in FI-MV–immunized mice with GC B cells first detected on day 12 and reaching their maximum (6.95%) on day 20. In addition to the delayed development, FI-MV–induced GCs resolved later (day 56) than VEE/SIN-H-induced GCs (day 30), showing similarity to SRBC-induced GCs (day 80). GC B cells were detected in the spleens of SRBC–immunized mice but not VEE/SIN-H or FI-MV–immunized mice (data not shown).

### Antibody-secreting cells specific for MV are present in short-lived and long-lived compartments

**Figure 4:**
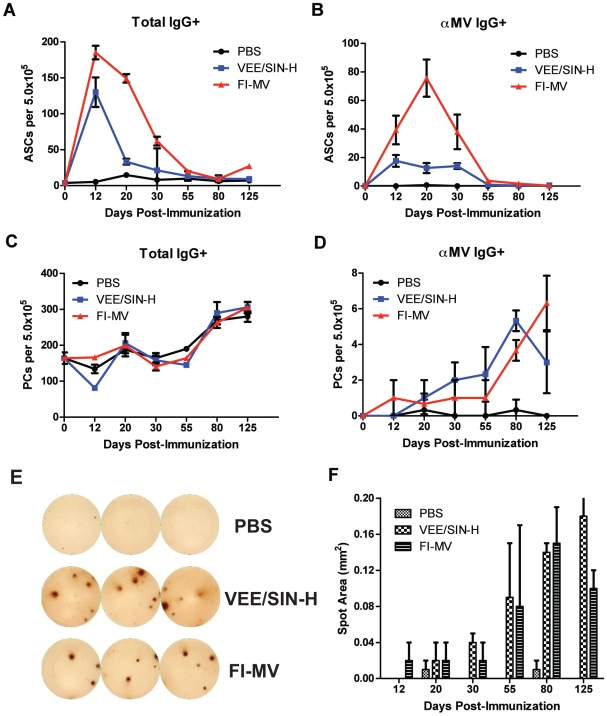
Development of antibody-secreting cells in draining lymph nodes and bone marrow after immunization. At various times after immunization, cells from draining popliteal LNs and bone marrow were collected and analyzed by IgG ELISpot. Total IgG-secreting cells were measured in the draining LNs (**A**) and the bone marrow (**C**), in addition to MV-specific ASCs (**B and D**). ELISpot plate images of bone marrow aspirates assayed for MV-specific ASCs at day 80 after immunization (**E**). The spot area for MV-specific IgG ASCs from the bone marrow at different times after immunization (**F**). Wells were loaded with 5.0×10^5^ unfractionated bone marrow cells. Assays were performed in triplicate (error bars represent S.D. of assay replicates) with cells pooled from 3 mice.

Antibody-secreting cells (ASCs) are generated in secondary lymphoid tissues and may or may not acquire the ability to mature into long-lived plasma cells and home to the bone marrow [63], [64]. Total IgG ASCs increased in dLNs after immunization with a peak at day 12 for both groups (Fig. 4A). MV-specific ASCs peaked at day 20 for FI-MV and peaked and then plateaued between days 12–40 for VEE/SIN-H (Fig. 4B). At day 20 the dLN ASC response to FI-MV was 6-fold greater than it was for VEE/SIN-H. Few ASCs were detected in the spleen for either vaccine (data not shown). In the bone marrow, numbers of IgG-secreting cells did not change (Fig. 4C), but small numbers of MV-specific ASCs began to appear by day 20 in both groups (Fig. 4D, E). The amount of MV-specific antibody secreted from individual bone marrow plasma cells increased through day 125, as indicated by the spot size, for VEE/SIN-H-immunized mice and through day 80 for FI-MV-immunized mice (Fig. 4F).

### Specificity of the B cell response to individual MV proteins

**Figure 5:**
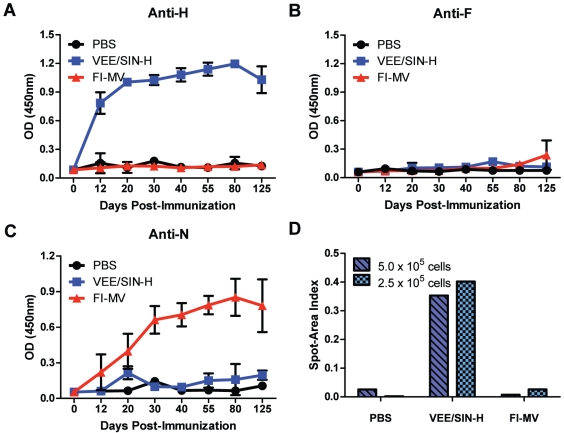
Protein-specific antibody responses to vaccine antigens. Serum IgG specific for MV H (**A**), F (**B**) and N (**C**) proteins over a 125-day time course measured by EIA. H-specific ASCs from the bone marrow measured by ELISpot at day 125, represented as an index of total spot number multiplied by spot area (**D**). Serum antibody data points represent the mean +/− S.D. of 3 individual mice. IgG ELISpot data are generated from cells pooled from 3 mice.

To determine why FI-MV–immunized mice did not develop neutralizing antibody (Fig. 1E) despite seroconversion (Fig. 1A), the specificity of serum IgG for individual MV proteins (H, F and N) was determined (Fig. 5). As expected, VEE/SIN-H–immunized mice showed a robust H-specific response and no response to F or N. FI-MV-immunized mice developed no detectable H-specific (Fig. 5A) or F-specific (Fig. 5B) IgG, but did develop antibody to N (Fig. 5C). At day 125 after immunization, plasma cells in the bone marrow secreting H-specific antibody were assessed (Fig. 5D). VEE/SIN-H-immunized mice had substantial numbers of plasma cells secreting antibody to H, while no H-specific plasma cells were detected in the bone marrow of FI-MV–immunized mice.

### MV-specific T cell responses in the draining lymph nodes

**Figure 6:**
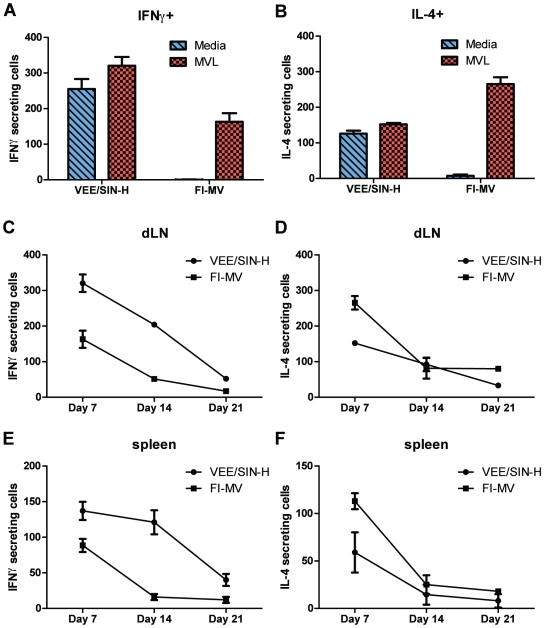
Measles virus-specific T cell responses after immunization. IFN-γ (**A**) and IL-4–secreting cells (**B**) from the dLN were measured at day 7 after immunization with VEE/SIN-H or FI-MV. Media-only stimulation represents *ex vivo* spontaneous secretion. This value has been subtracted from the MVL stimulation values. At days 7, 14 and 21 after immunization, cells from dLNs (**C, D**) and the spleen (**E, F**) were evaluated for IFNγ and IL-4 secretion after *ex vivo* stimulation with MVL antigen in ELISpot assays. Spot-forming cells are per 5×10^5^ total cells. Assays were performed in triplicate (error bars represent S.D. of assay replicates) with cells pooled from 3 mice.

To compare T cell responses, draining lymph node (LN) cells were assayed by ELISpot for IFN-γ and IL-4-producing cells directly *ex vivo* and after stimulation *in vitro* with MV antigen 7, 14 and 21 days after immunization (Fig. 6). During the peak response at day 7, VEE/SIN-H, but not FI-MV, induced substantial numbers of IFN-γ (Fig. 6A) and IL-4 (Fig. 6B) spot-forming cells (SFCs) that were detected directly *ex vivo*. Stimulation with MV increased the numbers of cells from VEE/SIN-H–immunized mice producing IFN-γ and IL-4 and induced IFN-γ and IL-4 expression by cells from FI-MV–immunized mice. dLN cells from VEE/SIN-H–immunized mice predominantly produced IFN-γ with IFN-γ/IL-4 SFC ratios of 2.2 at day 7 and 2.1 at day 14 (Fig. 6C, D). In contrast, dLN cells from FI-MV–immunized mice predominantly produced IL-4 with an IFN-γ/IL-4 SFC ratio of 0.61 at day 7 and 0.63 at day 14. Responses of splenocytes to MV antigen stimulation were similar in pattern to those observed in the dLN, but lower in magnitude (Fig. 6E, F).

**Figure 7:**
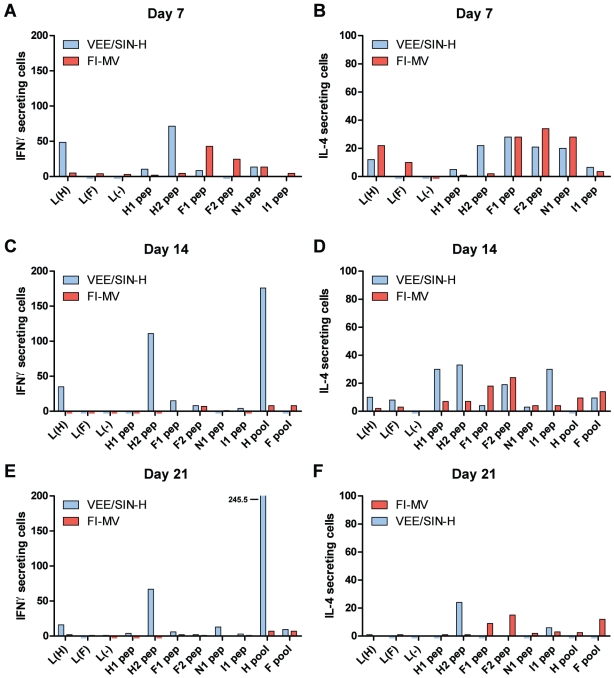
Protein-specific T cell responses after immunization. Cytokine secretion as a readout for recognition of class-II restricted CD4^+^ T cell MV epitopes for H (H1, H2), F (F1, F2) and N (N1) proteins, as well as complete peptide pools for H and F proteins, was measured for IFN-γ (**A, C, E**) and IL-4 (**B, D, F**) in cells from the dLN by ELISpot at 1, 2 and 3 weeks after immunization. Negative controls were an irrelevant influenza HA peptide (I1 - I-E^d^) and media-only and ConA-stimulated cells (not shown) served as a positive control (n.d. = not determined). Negative controls (media-only) were subtracted to discount cells that spontaneously produced cytokines and to emphasize antigen-specific reactivity. Spot-forming cells are per 5×10^5^ total cells. Assays were performed in triplicate (error bars represent S.D. of assay replicates) with cells pooled from 3 mice.

To better characterize the CD4^+^ T cell response, responses to known class II-restricted (I-E^d^) CD4^+^ T cell epitopes for H, F and N proteins [65]–[70], as well as L cell lysates and peptide pools, were assessed (Fig. 7). VEE/SIN-H–immunized mice produced IFN-γ in response to the H2 peptide, H L cell lysate and H peptide pool at all time points examined while H-specific IFN-γ production by FI-MV mice was negligible (Fig. 7A, C, E). However, FI-MV–immunized mice produced IL-4 in response to stimulation with both H- and F-containing L cell lysates 7 days after immunization (Fig. 7B). The response to H was 8.5% of the IL-4 response after MVL stimulation at the same time point (Fig. 6B). F-specific secretion of IL-4 was stimulated by individual class II peptides F1 and F2, as well as the F peptide pool through day 21 (Fig. 7B, D, F) and F-specific IFN-γ secretion at day 7 (Fig. 7A), represented 26.5% of the IFN-γ-secreting cells stimulation by MVL.

### B cell recall responses to H

**Figure 8:**
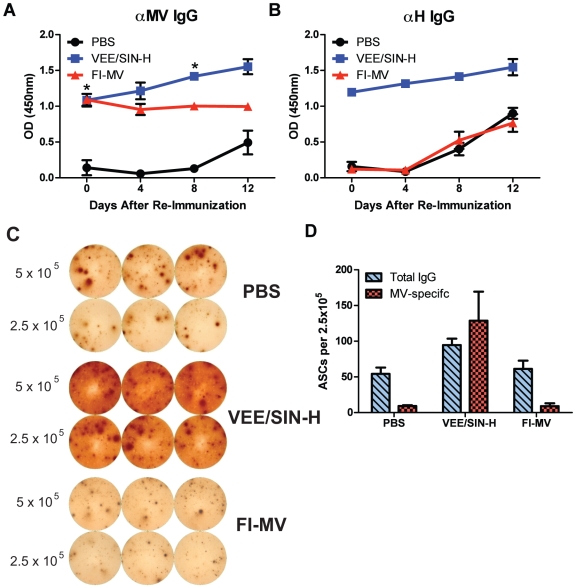
Hemagglutinin-specific antibody-secreting cell recall responses. Mice immunized with FI-MV, VEE/SIN-H or mock-immunized with PBS were given VEE/SIN-H at day 81 after primary immunization, bled at days 4 and 8 post-secondary immunization and sacrificed at day 12. EIAs were performed to measure serum antibody with reactivity against MVL (* *P*<0.05; Student's *t* test) (**A**) and H (**B**) induced in the recall response up to 12 days after administering VEE/SIN-H. ASCs in the draining LNs were assayed for total IgG and MV-specific antibody secretion by ELISpot (**C, D**) 12 days after secondary immunization. Serum antibody data points represent the mean +/− S.D. of 3 individual mice. IgG ELISpots were performed in triplicate (error bars represent S.D. of assay replicates) with cells pooled from 3 mice.

To assess the development of H-specific memory B cells, VEE/SIN-H was administered 81 days after initial immunization with FI-MV, VEE/SIN-H or PBS. VEE/SIN-H–immunized mice showed increased production of anti-MV antibody (Fig. 8A) and anti H antibody (Fig. 8B) by 4 days after a secondary immunization with VEE/SIN-H (*p*<0.05). FI-MV–immunized mice did not show an increase in anti-H titer until day 8 and the kinetics and magnitude were similar to the PBS control mice (Fig. 8B). At day 12, dLNs were collected and assayed for MV-specific ASCs (Fig. 8C, D). Mice immunized with FI-MV and then boosted with VEE/SIN-H had MV-specific ASCs at a level similar to that of control mice initially injected with PBS, while VEE/SIN-H-immunized mice had a large number of MV-specific ASC comparable to the numbers of IgG ASC.

## Discussion

Measles remains a worldwide public health concern and is a particular threat to health in early life. Recent efforts have focused on developing a vaccine that will circumvent the maternal antibody barrier for immunizing young children, while avoiding problems encountered with FI-MV, and on new routes of delivery for the current vaccine. To better understand the problems with responses to FI-MV, as well as to obtain information on the immune response to a promising new vaccine, we compared the responses of mice to FI-MV and VEE/SIN-H. The immune responses to FI-MV were characterized by a slow induction of GC formation, low avidity MV-specific IgG with IgG1>IgG2a, high levels of antibody-secreting B cells in the dLNs, and T cells that produced more IL-4 than IFN-γ in response to stimulation with MV. VEE/SIN-H rapidly induced GC formation in the dLN, high avidity MV-specific IgG with IgG2a>IgG1, neutralizing antibody and T cells that produced IFN-γ and IL-4 both spontaneously *ex vivo* and in response to stimulation with H. FI-MV did not induce H-specific antibody, memory B cells or plasma cells and thus did not induce neutralizing antibody. Instead, FI-MV induced a low avidity antibody to N. Thus, these measles vaccines differed in antibody specificity, isotype, avidity, local B cell responses and T cell cytokine profiles.

The most important correlate of vaccine-induced protection from measles is the presence of high avidity neutralizing antibody at the time of exposure to wild type MV [37]. This study has shown that vaccination of mice with the chimeric alphavirus replicon particle vaccine VEE/SIN-H resulted in a LN environment that promoted avidity maturation of antibody to H and the production of ASCs that homed to the bone marrow, a site of sustained production of antibody for humoral memory [64], [71]–[75].

FI-MV–immunized mice developed a poor response to the H protein. Antibody to H was not detected by EIA or by PRN and T cells did not respond to H peptide stimulation with production of IFN-γ or IL-4. Furthermore, boosting of FI-MV-vaccinated mice with VEE/SIN-H did not elicit an anamnestic B cell response to H. Lack of antibody to the H envelope glycoprotein is consistent with the absence of neutralization capacity. FI-MV induced abundant antibody to N, a viral protein that resides in the interior of virion and is highly structured [76]. These differences in viral protein immunogenicity may reflect differential stabilization by formalin and methylene bridges generated by formaldehyde polymers [77] may alter available B cell epitopes. Highly ordered structures such as N may allow for more epitope-preserving formalin crosslinking. Early studies of immune responses to inactivated measles vaccine in humans reported a lack of antibody to F [78], but antibodies to H were detected in vaccinees and also in monkeys immunized with FI-MV. However, these antibodies waned quickly leaving individuals susceptible to MV infection [33], [34]. MV envelope glycoproteins are relatively labile vaccine antigens and lack of an H response suggests denaturation by the combination of formalin-treatment, alum precipitation and time.

The adjuvant activities of alum and alphavirus particles are distinct. Alum-precipitated antigens are taken up by dendritic cells (DCs) where they activate the NLRP3 inflammasome for caspase 1-dependent production of IL-1β, possibly through increasing local concentrations of uric acid [79]. Chemokines CCL2 and CCL11 are produced within hours resulting in attraction of inflammatory cells to the site. Early production of IL-4 inhibits the differentiation of Th1 cells resulting in a Th2-biased response that supports B cell and antibody responses [80], [81]. However, it is unclear why this Th2-dominant environment for B cell differentiation resulted in an inferior antibody response that was not maintained in humoral memory when the vaccine was given to humans.

In contrast, alphavirus replicon particles target DCs [82] and feature viral molecular patterns with intrinsic adjuvant effects [53], [83]. Replicons expressing heterologous proteins or null-replicons co-administered with protein antigens induce the rapid local production of cytokines and chemokines including IFN-β, IL-5, IL-6, TNF-α, CCL4, CCL5 and CXCL10 [54], [84], [85]. Interestingly, these vaccines induce production of IgA and CD8^+^ T cells that appear at mucosal sites [52], [54], [86], [87], properties likely to be of benefit for a vaccine against measles.

A primary difference between the vaccines was in the maturation of antibody avidity. VEE/SIN-H and FI-MV initiated GC responses of similar magnitude. GCs are the sites of somatic hypermutation of variable region DNA and selection for B cells that possess antigen receptors with high affinity [88]. FI-MV induced a large extrafollicular B cell response, but there was no evidence of a T-independent response, as no MV-specific IgG3 was detected and antibody responses of XID mice were similar to those of control mice (data not shown). GCs in the dLNs of FI-MV-immunized mice peaked approximately 7 days later than GCs in the lymph nodes of VEE/SIN-H and SRBC–immunized mice. This late time course is observed with other alum-precipitated vaccines and perhaps reflects the quality of the T cell response or the “depot effect” commonly attributed to this adjuvant [89]–[91]. GC reactions for most T-dependent antigens peak around 10–12 days [92], but instances of long-lived GCs have been linked to persisting antigen [93]. The avidity of VEE/SIN-H–induced IgG increased beginning at day 36, well after the peak of GC formation. This lag may be due to the dilution of emerging avid clones by earlier short-lived ASCs [94] secreting unmatured IgG or to continued avidity maturation at sites outside GCs.

We conclude that a primary reason for the failure of FI-MV is likely to be the poor preservation of the immunogenicity of the MV H protein. FI-MV also induced poor avidity maturation of antibody to N and elicited a type 2-skewed T cell response. In contrast, VEE/SIN-H induced a robust and balanced T and B cell response to the MV H protein that resulted in durable production of affinity-matured neutralizing antibody. Future studies of this vaccine will require consideration of the addition of other MV antigens, in addition to manufacturing, cost, safety and immunogenicity in humans.

## Materials and Methods

### Mice, vaccines and immunization

Eight-to-ten week-old female BALB/c (Charles River Laboratories, Wilmington, MA), mice were used. Alum-precipitated FI-MV prepared in the 1960s (Pfizer, Terre Haute, IN; gift of Albert Kapikian, National Institutes of Allergy and Infectious Diseases, Bethesda, MD) was given at a dose of 50 µL per mouse. A chimeric VEE/SIN-based replicon particle vaccine [48] engineered to express MV Edmonston strain H [55] was diluted in 40 mg/mL lactose/PBS and given at a dose of 1.25×10^6^ particles, previously shown to be optimal for alphavirus replicon particles expressing H [11]. Control mice were injected with PBS or with 1–5×10^9^ PBS-washed SRBCs, (Colorado Serum Company, Denver, CO). All immunizations were administered subcutaneously (standard for measles vaccines) in both hind feet to facilitate access to the dLNs. At various times after immunization, mice were anesthetized with isoflurane (Abbott Laboratories, North Chicago, IL) and blood, draining popliteal LNs, spleens and bone marrow were collected. Mice were maintained under specific pathogen-free conditions and used in accordance with protocols approved by the Johns Hopkins University Animal Care and Use Committee.

### MV Antigens

For total MV protein, a lysate of MV-infected Vero cells (MVL) (Advanced Biotechnologies Inc., Columbia, MD) was used. For MV H and F envelope proteins, lysates were prepared from L929 murine fibroblasts expressing either H or F [95] (a gift from Fabian Wild, Pasteur Institute, Lyon, France). The BaculoDirect™ Baculovirus Expression System (Invitrogen, Carlsbad, CA) was used to generate full-length N protein from infected Sf9 insect cells. All MV proteins were from the Edmonston strain and were used in immunoassays as clarified lysates.

Twenty-mer peptides (with 11 amino acid overlaps) covering the sequences of H and F were synthesized using solid-phase peptide chemistry by the JHU Synthesis and Sequencing Facility and peptides for each protein were pooled. In addition, individual peptides corresponding to previously mapped MHC-II-restricted (I-E^d^) epitopes of MV H, F and N proteins [65]–[69] were synthesized: H – LYKSNHNNVYWLTIP (aa 446–460; H1), YSPGRSFSYFYPFRL (aa 546–560; H2); F – LLGILESRGIKARIT (aa 256–270; F1), PVVEVNGVTIQVGSR (aa 421–435; F2); N – YAMGVGVELEN (aa 335–345; N1). All peptides were based on the MV Edmonston sequence.

### Antibody Assays

To measure MV-specific IgG, 96-well Maxisorp™ ELISA plates (Nalge Nunc International, Rochester, NY) were coated with MVL, lysates of L cells expressing H or F or with baculovirus-generated N diluted in 50 mM sodium bicarbonate buffer, pH 9.6. Wells were coated overnight at 4°C, plates were blocked with 2% non-fat dry milk and individual serum samples were diluted ten-fold in 1% non-fat dry milk for analysis. MV-specific binding was detected with horseradish peroxidase (HRP)-labeled goat anti-mouse IgG, IgG1, IgG2a or IgG3 (Southern Biotech, Birmingham, AL) and 3,3′, 5,5′-tetramethylbenzidine (Sigma) as the enzymatic substrate. For avidity measurements, the enzyme immunoassay (EIA) was modified to include a 0-3.5M ammonium thiocyanate (NH_4_SCN) wash in 0.5M steps to dissociate bound IgG [96], [97]. The avidity index is the concentration of NH_4_SCN at which 50% of the antibody was eluted.

Neutralizing antibody was measured by plaque reduction (PRN) as previously described [98] using the Chicago-1 strain of MV for infection of Vero cells to calculate 50% neutralization titers. Data are reported as geometric mean titer for 3 animals at each time of sampling. The assay was run in triplicate for each sample.

### Histology

Freshly harvested, OCT-embedded draining LNs were cryosectioned to 10µm thickness on a Microm HM-500 cryostat (Walldorf, Germany) and fixed in cold acetone. Sections were blocked with 10% normal rat serum (Chemicon, Temecula, CA) followed by staining with PNA-biotin (10 µg/mL) (Vector Laboratories, Burlingame, CA) and rat anti-mouse IgD-PE (1 µg/mL) (Southern Biotech) overnight at 4°C. PNA^+^ cells in GCs were identified with streptavidin conjugated to Alexa Fluor™ 488 (5 µg/mL, Invitrogen). Sections were mounted with Shur-Mount (EM Sciences, Ft. Washington, PA) and viewed under a Nikon E800 fluorescent microscope. Images of sections from each mouse were analyzed for GCs using SPOT Advanced™ software (Diagnostic Instruments, Sterling Heights, MI).

### Flow Cytometry

To identify GC B cells, draining LN cells were incubated with purified rat anti-mouse CD16/CD32 (5 µg/mL) (BD Biosciences) to block Fc receptors and then stained with Alexa Fluor™ 647-conjugated rat anti-mouse CD19 (2 µg/mL) (BD Biosciences) and FITC-conjugated PNA (Sigma) at 0.2 µg/mL in 0.1% BSA/PBS with 0.02% NaN_3_. Samples were analyzed on a FACSCalibur flow cytometer (BD Biosciences) and the data were evaluated using Flowjo™ software v8.7.3 (Tree Star, Ashland, OR).

### ELISpot Assays

96-well Multiscreen™ HTS HA Opaque ELISpot plates (Millipore, Billerica, MA) were used. Plates were coated with MV antigens (as described above) or with purified goat anti-mouse Ig (Southern Biotech) at 10 µg/mL and blocked in complete RPMI-10 media for 2 h at 37°C. Single-cell suspensions from dLNs or bone marrow were plated at various concentrations in fresh media and incubated for 8 h at 37°C, 5% CO_2_. Bone marrow aspirates were treated with RBC lysis buffer (Sigma) and washed prior to plating. After incubation, bound IgG was detected with HRP-labeled goat anti-mouse IgG (1∶5,000) (GE Healthcare, Piscataway, NJ) and developed with stable diaminobenzidine (DAB) (Invitrogen) and read on an ImmunoSpot™ plate reader (Cellular Technology, Shaker Heights, OH). The data were analyzed with ImmunoSpot™ 2.0.5 software.

For assays of IFN-γ and IL-4–secreting cells, ELISpot assays were performed as above using plates coated with purified rat anti-mouse IFN-γ or IL-4 capture antibodies (BD Biosciences) at 5 µg/mL. Biotin-conjugated rat anti-mouse IFN-γ or IL-4 detection antibodies (2 µg/mL) (BD Biosciences) and avidin-D-HRP conjugate (Vector Laboratories) were used for development. Cells were incubated for 48 h at 37°C, 5% CO_2_. *Ex vivo* culture stimulants for IFN-γ and IL-4 ELISpot assays included MVL (1∶100), L(H) lysate (1∶20), L(F) lysate (1∶20), H and F peptide pools at 1 µg/mL and individual MV peptides (H1, H2, F1, F2, N1) at 5 µg/mL (described above). Controls included an irrelevant I-E^d^-restricted peptide from the hemagglutinin of influenza A–KYVKQNTLKL (I1) [70] at 5 µg/mL, normal L cell lysate (L(-)) diluted 1∶20 and media alone, while concanavalin A-stimulated cells (5 µg/mL) (Sigma) served as a positive control.
